# Fault Diagnosis of a Rotor and Ball-Bearing System Using DWT Integrated with SVM, GRNN, and Visual Dot Patterns

**DOI:** 10.3390/s19214806

**Published:** 2019-11-05

**Authors:** Wen-Lin Chu, Chih-Jer Lin, Kai-Chun Kao

**Affiliations:** 1 Department of Mechanical Engineering, National Chin-Yi University of Technology, Taichung 41170, Taiwan; wlchu@ncut.edu.tw; 2 Graduate Institute of automation Technology, National Taipei University of Technology, Taipei 10608, Taiwan; karenkau9315@gmail.com

**Keywords:** support vector machines, general regression neural networks, diagnosis of malfunction, convolutional neural network

## Abstract

In this study, a set of methods for the inspection of a working motor in real time was proposed. The aim was to determine if ball-bearing operation is normal or abnormal and to conduct an inspection in real time. The system consists of motor control and measurement systems. The motor control system provides a set fixed speed, and the measurement system uses an accelerometer to measure the vibration, and the collected signal data are sent to a PC for analysis. This paper gives the details of the decomposition of vibration signals, using discrete wavelet transform (DWT) and computation of the features. It includes the classification of the features after analysis. Two major methods are used for the diagnosis of malfunction, the support vector machines (SVM) and general regression neural networks (GRNN). For visualization and to input the signals for visualization, they were input into a convolutional neural network (CNN) for further classification, as well as for the comparison of performance and results. Unique experimental processes were established with a particular hardware combination, and a comparison with commonly used methods was made. The results can be used for the design of a real-time motor that bears a diagnostic and malfunction warning system. This research establishes its own experimental process, according to the hardware combination and comparison of commonly used methods in research; a design for a real-time diagnosis of motor malfunction, as well as an early warning system, can be built thereupon.

## 1. Introduction

Automated inspection is growing in order to keep pace with the rapid development of production technology. This has become essential for precision machining and the proper control of product quality [1,2]. The automated inspection industry has become very important in the many and diverse industries where automation has been introduced [3]. Not only does it increase productivity by reducing production losses [4], but it also raises safety in intelligent factories [5,6]. For example, maintenance and repair can be done immediately—an abnormality is found in the status of a motor by automatic diagnosis. Accelerometers are commonly used to detect the functional status of motors or other moving parts of machines, and the analysis of the one-dimensional signal from an accelerometer has become a crucial matter in this respect. However, it is rather difficult to determine if a signal collected from an accelerometer, attached to a motor or machine, originates from normal behavior or an abnormality. This has resulted in much work being done on spectrum analysis as a major procedure for the assessment of the signals and to increase computational speed [7]. The fast Fourier transform (FFT) [8] has long been the principal means for signal processing in the frequency domain [9]. To understand the timing of frequency variation from within, the idea of wavelets needs to be introduced. When Goupillaud et al. [10] analyzed seismic waves, they found traditional FFT could not meet the expected outcome, and the wavelet transform (WT) [11] was born. Daubechies [12] defined a hierarchical and orthogonal discrete wavelet transform (DWT) that can be used for the analysis of signals, as well as for compression and to remove noise. After either frequency or wavelet domain analysis, signal features can be obtained by calculation, and they can be used as a model for teaching the classifier. Machine learning is a subset of artificial intelligence (AI), where the patterns and behaviors are always reached by learning from information and experience to achieve the purpose of AI. The teaching methods used for machine learning can be either supervised or unsupervised. The support vector machine (SVM) is a supervised learning algorithm [13,14] derived in connection with neural networks. It is a learning machine based on statistical learning theory. This was first proposed by Vapnik [15], who worked on pattern classification and nonlinear regression. SVM performs very well with small samples and for nonlinear and high-dimensional identification. It has rather broad applications, for example, in handwriting recognition, human-facial and image recognition, text classification, three-dimensional object identification, and so on. SVM defines a hyperplane with maximum margin in a space and exploits this hyperplane to separate samples into two different classes. In addition to SVM, other neural networks have also flourished. In 1998, Specht [16] proposed the probability neural network (PNN), a one-pass learning process compatible only with classification, because it cannot solve the problem of continuous variables. For this, Specht [17] proposed the general regression neural network (GRNN) and radial basis network (RBN) in 1991. GRNN not only provides solutions unavailable from PNN but also learns; it can also provide answers to both linear or nonlinear problems with the same efficiency. However, a feed-forward neural network, convolutional neural network (CNN), is capable of resolving many sorting problems at high dimensions [18,19] The basic structure of the network consists of one or several convolution layer(s) and pooling layer(s), as well as full-connection layer(s); these enhance the relativity between pattern recognition and data. A better outcome can be achieved with CNN on signals data, such as images and sounds. The related methods from Zhu et al. [20,21] that propose a CNN model for symmetrized dot pattern (SDP) image recognition can then be applied to rotor vibration for fault diagnosis.

There are two main topics in this study: motor control and the measurement system. A proportional-integral-derivative (PID) controller was used to regulate the rotational speed of a servomotor, and an accelerometer was used to measure the vibration signals from the motor. A myRIO card was used to capture the signals after signal conditioning for transmission to a PC terminal. In the subsequent analysis, two methods were used: The first carried out signal spectrum analysis and picked the optimum frequency band for feature calculation. This was then processed by SVM and GRNN and served as the input data of the teaching model. This model was used as a diagnostic basis for motor-bearing malfunction. The second was to render the vibrations pictorially. All kinds of malfunction vibrations were input into CNN for further classification, as well as to make comparisons of the classifier functions among SVM, GRNN, and CNN.

## 2. Materials and Methods

The main purpose of this project was to develop a method for the diagnosis of malfunction in the ball bearings of an electrical motor and to use vibration signals generated by bearing rotation, to determine the presence of abnormality. A malfunction simulation system was configured in the scheme to introduce malfunction, explanations of which are in the experimental-setup section. The frequency of ball-bearing vibration signals and the process under wavelet domain are explained in the analysis and process-of-vibration-signals section. A discussion of the formula for computing the feature is introduced in the motor-signal-features section. Machine learning, teaching, and classification by CNN, as well as visualization of the signals, are fully elucidated in the teaching-and-classification-of-CNN and machine-learning (ML) sections.

### 2.1. Experimental Setup

**Figure 1:**
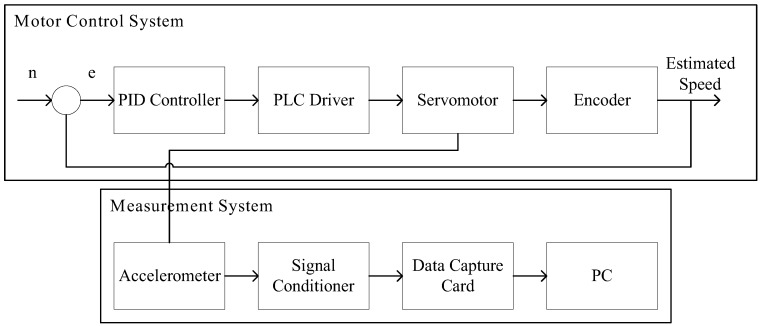
System structure.

The major structure of this system includes a motor control system and measurement system. The motor control system manages to bridle a motor through a PC and BeckHoff controller and uses a PID controller algorithm. A set of human-machine interface (HMI) is built into the PC to monitor motor status in real time. As for the measurement system, it can capture the signals from the accelerometer through NI myRIO embedded system, as well as signal conditioner. Both the motor control system and measurement system are applied to the motor measurement platform developed in the lab, which is shown in Figure 1; the related hardware specifications listed in Table 1.

The BeckHoff controller is an embedded PC installable in DIN guide rails which includes a CX-series (CX5140) PC and an I/O module. This series features low power consumption and a fan-free design. It has one 4-core Intel^®^ Atom™ CPU (1.91GHz) two independent Gigabit Ethernet ports, four USB2.0 ports, and one DVI-I port. The software included is the TwinCAT (Windows Control Automation Technology) system that was developed by BeckHoff and can interface with a PLC (Programmable Logic Controller) and NC/CNC. This PC is a fully functional controller. TwinCAT is compiled as a Run-Time system, which can run many programs, conduct diagnoses, and is fully configurable.

The drive module for the servomotor is an EL2111, which has an adequate capacity for the purpose. EtherCAT (Ethernet for Control Automation Technology) was used for communication and the client/server (C/S) model only needed a standard Ethernet card to connect to the host controller of the master terminal. The main application is automation, and a field-bus of an industrial control system (ICS) was used. The system has wide compatibility and is expandable. It is easy to operate and synchronizes very well. No specific hardware interface card was not needed, and connection was made using an Ethernet controller and a network port.

**Figure 2:**
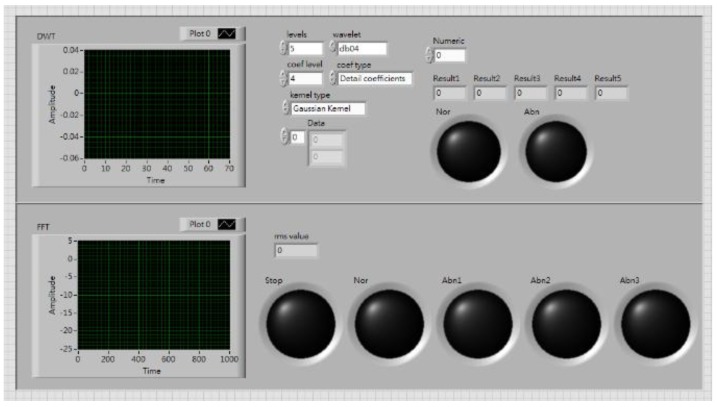
GUI screen.

**Figure 3:**
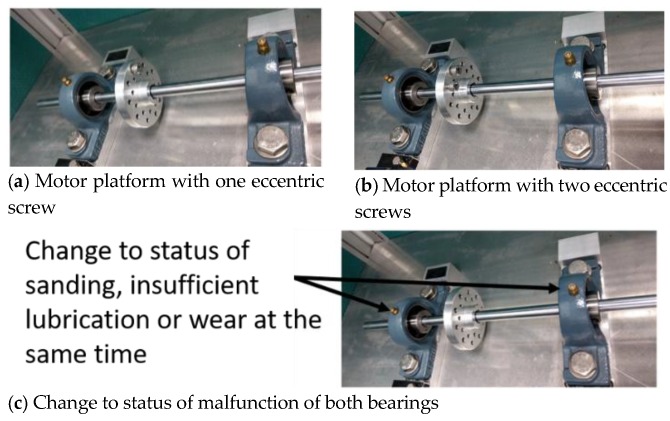
Malfunction simulation schematic.

LabView captured the signals from the myRIO and transferred them to the PC terminal, for computation and classification through global variables. The graphical user interface (GUI) is shown in Figure 2. Six status conditions were used: (1) normal; (2) motor platform with one eccentric screw; (3) motor platform with two eccentric screws; (4) sanded ball bearings; (5) insufficient lubrication; and (6) worn ball bearings (see Figure 3).

### 2.2. Analysis and Process of Vibration Signals of Ball Bearings

**Figure 4:**
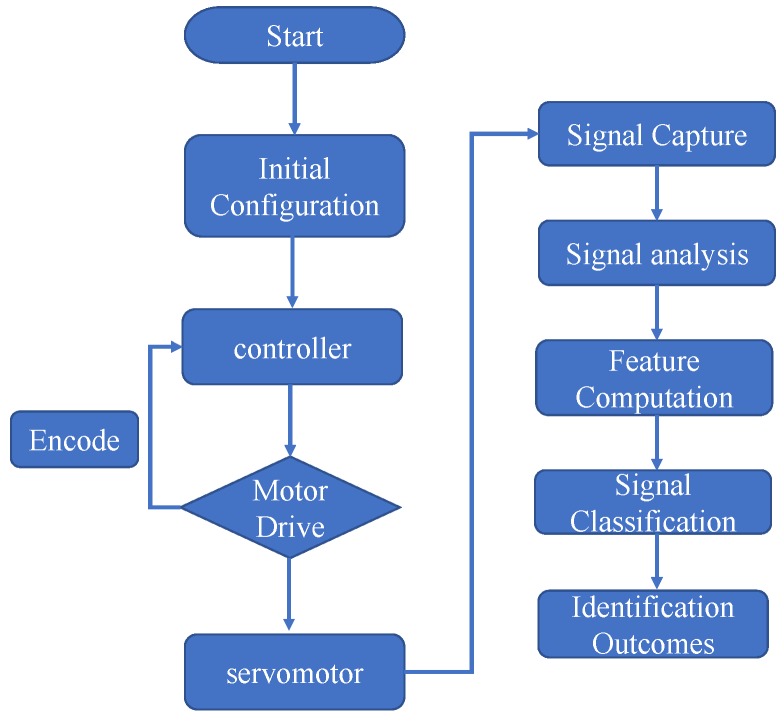
Malfunction simulation.

In this section, the principal analysis and processing of signals is introduced. As explained in the experimental-setup section, all the signals were transferred to the PC terminal, where the features were transformed either in the frequency domain or as discrete wavelets, before analysis. The results of the diagnoses, using the different classifiers, are shown in Figure 4.

The vibration signals collected from various time domains and with different motor status are mixed with lots of noise from different frequency bands, and this presents a problem. To find useable features in the time domain, the signals need to be analyzed and treated before features can be selected for calculation. In this study, the vibration signals were first transformed in the frequency domain by FFT, a linear integral transform. It can transform the signals between time domain and frequency domain, and it can also decompose the time-domain signals into the superposition of sine waves from many different frequencies. The formula is shown as Equation (1):

(1) $$F\left(w\right)=\int_{-\infty }^{+\infty }f\left(t\right)e^{-iwt}dt$$

where f(t) is the input signal of continuous time, w is the angular frequency, and t is time. However, if the signals are represented by a form of discretion and periodicity in both the time domain and the frequency domain, it is a discrete Fourier transform (DFT). If the volume of data is large, the calculation of DFT can be enormous, and computation time will be long, where the computational complexity will be $O\left(n^{2}\right)$. The fast Fourier transform (FFT) is a quick approach to the calculation of DFT. When dealing with general signals, the computation volume of FFT is much less than that of DFT, where computational complexity is O(nlogn). FFT has excellent analytical performance for stationary signals where frequency does not change over time, but it cannot be used for time variation analysis of nonstationary signals. Locality does not exist, and it is only compatible with stable signals.

To provide a solution to this problem, Gabor proposed the short-time Fourier transform (STFT), which is a window function, and the formula is as in [22]:

(2) $$S\left(w,\tau \right)=\int_{-\infty }^{+\infty }f\left(t\right)g_{a}\left(t-\tau \right)e^{-iwt}dt$$

where $g_{a}\left(t-\tau \right)$ is the window function, and τ is the translation parameter. The window function can split up the signals into many small time intervals, where the Fourier transform $\left(\int f\left(t\right)e^{-iwt}dt\right)$ can be done in a local time domain (t−τ), the position of the window function $g_{a}\left(t-\tau \right)$ is changed along with the translation parameter τ, and S(w,τ) can be obtained for each time interval, representing the intensity of a signal at a specific time and frequency. The window function provides a solution to the Fourier transform local signal problem, and the window dimensions in such a function are fixed. A large window will give good time resolution, but the frequency resolution will be poor, so a big window is suitable for the analysis of low-frequency signals. On the other hand, a small window gives poor time resolution but good frequency resolution and is suitable for the analysis of high-frequency signals.

Because the window dimension in a Fourier transform with a short time interval is fixed, analysis with good resolution of both time and frequency is not possible. However, the wavelet transform (WT) contains the characteristics of both dilation and translation, where the foundation transformed through the wavelet can be changed through its function. The formula is shown as Equation (3):

(3) $$\varphi_{a,\tau }\left(t\right)=\frac{1}{\sqrt{\left|a\right|}}\varphi \left(\frac{t-\tau }{a}\right)$$

where φ(t) is the mother wavelet, a is the scale parameter, and τ is the translation parameter. The scale parameter, a, in this wavelet transform formula can be extended or compressed based on the mother wavelet. A larger scale parameter, a, allows for a more extensive mother wavelet to be applied to the signal with a higher frequency. On the other hand, a smaller scale parameter, a, means a more compressed mother wavelet at a lower frequency. In addition, the translation parameter can permit the mother wavelet to be translated over the time axis, where the mother wavelet, φ(t), must comply with conditions from Equations (4) to (6). In Equation (4), the mother wavelet oscillates, while, in Equations (5) and (6), most of the function energy is restrained within a limited time, where the amplitude decays from both the positive and negative directions to zero.

(4) $$\int_{-\infty }^{\infty }\varphi \left(t\right)dt=0$$

(5) $$\int_{-\infty }^{\infty }{\left|\varphi \left(t\right)\right|}^{2}dt=1$$

(6) $$\int_{-\infty }^{\infty }\left|\varphi \left(t\right)\right|dt<\infty$$

The computational complexity can be reduced through discretization among the continuous parameters transformed by the wavelet, such as scale parameter, a, and translation parameter, τ. Thus, we adjust those two parameters, a and τ, in Equation (3), respectively, to $a=a_{0}^{m}$ and τ=na, wherein m,n∈Z, and $a_{0}\neq 1$. After this adjustment, the new definition of wavelet function is as in Equation (7):

(7) $$\varphi_{m,n}\left(t\right)=a_{0}^{-\frac{m}{2}}\varphi \left(a_{0}^{-m}t-n\right)$$

If $a_{0}=2$, then it is a dual wavelet function (Dyadic Wavelet), which is shown as Equation (8):

(8) $$\varphi_{m,n}\left(t\right)=\frac{1}{\sqrt{2^{m}}}\varphi \left(\frac{t-n}{2^{m}}\right)$$

After this, it is only necessary to select a proper value of *m* to analyze the signals. The discrete wavelet transform is shown as Equation (9):

(9) $$DWT_{f}\left(m,n\right)=\frac{1}{\sqrt{2^{m}}}\sum_{k}f\left(k\right)\varphi \left(\frac{k-n}{2^{m}}\right)$$

where k is the calculation pointer, and f(k) is the coefficient of wavelet transform, while discrete wavelet transforms the continuous signals, and only the two variables are discrete, the scale parameter, a, and the translation parameter, τ.

The discrete wavelet transform decomposes the original signals through both high-pass and low-pass filters (the wavelet and scaling functions), and the decomposed signals are detailed and approximate. The filtering process doubles the volume of the signals, and data sampling is carried out after it is completed.

There are various wavelet functions that can be chosen for diverse purposes. Daubechies Wavelet is an orthogonal wavelet featuring hierarchy. It is primarily used for the detection of wavelet transformation, and its wavelet clans range from db2 to db10, where the numerics indicate vanishing moments. The larger the numeric, the smoother the wavelet curve. The sampling frequencies used in this study were 1000 and 3000 Hz, db4 in the Daubechies clan. The motor signals are computed by five-layer decomposition, and the resolved frequencies obtained for each layer are as shown in Table 2.

**Figure 5:**
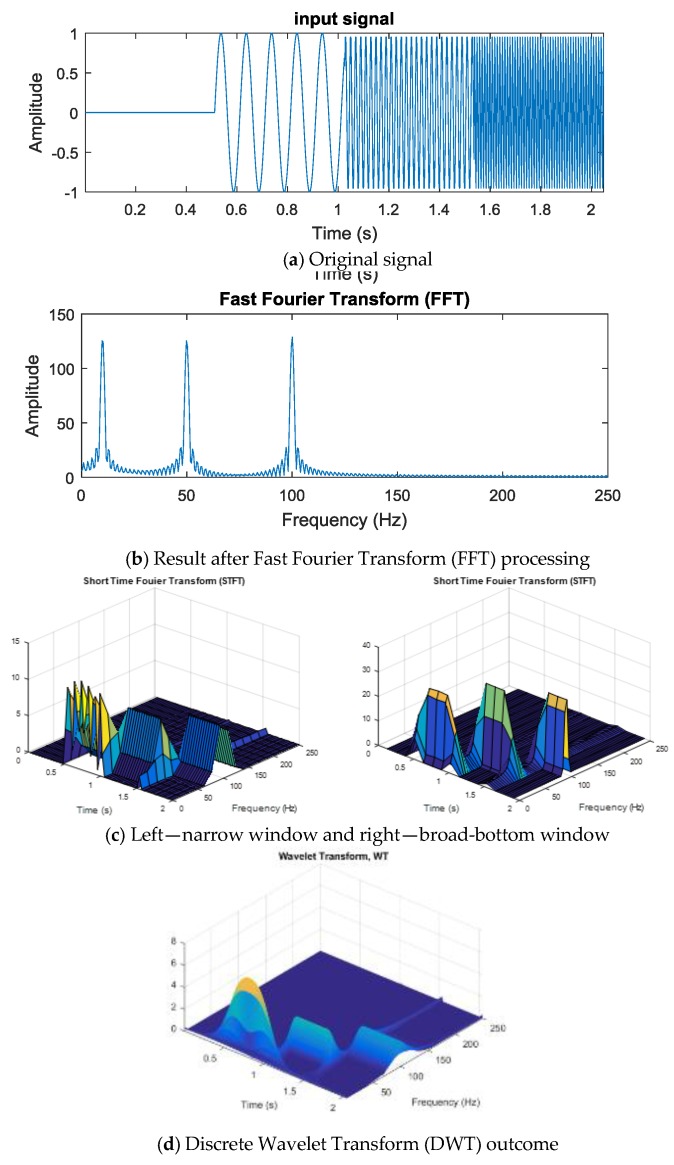
(**a**) Original signal; (**b**) results after FFT; (**c**) narrow-window, and broad-bottom-window; and (**d**) DWT outcome.

FFT, STFT, and DWT were introduced in the preceding paragraphs, and the three respective outcomes are now presented in Figure 5. The input chirp signal is shown in Figure 5a. It is obvious from Figure 5b that FFT cannot present the linearity of a chirp signal. Furthermore, an apparent dilemma can be seen in selecting an appropriate window of analysis on time and frequency for STFT in Figure 5c. Figure 5d shows the wavelet transform that can be finally acquired.

### 2.3. Feature of Motor Signal

Since it is not possible to directly identify the signals with various classifications which have been processed through discrete wavelet transform by using the naked eye, it is necessary to find a way to process the computation of the feature for those signals being transformed via the wavelet. Six feature methods were used in this study: maximum, minimum, mean, range, standard deviation, and median absolute deviation, and the formulas are shown as Equations (10)–(14):

(10) $$X_{N,\max}=\underset{0\leq n\leq N}{\max}\left(X_{N}\left(n\right)\right)$$

(11) $$X_{N,\min}=\underset{0\leq n\leq N}{\min}\left(X_{N}\left(n\right)\right)$$

(12) $$X_{N,mean}=\frac{1}{N}\sum_{n=1}^{N}\left|X_{N}\left(n\right)\right|$$

(13) $$X_{N,range}=X_{N,\max}-X_{N,\min}$$

(14) $$\begin{array}{l} X_{N,\sigma }=\sqrt{\frac{1}{\tilde{N}}\sum_{n=1}^{\tilde{N}}{\left(X_{N}\left(n\right)-X_{N,\mu }\right)}^{2}}n^{th}\\ \mathrm{Wherein}\;X_{N,\mu }=\frac{1}{\tilde{N}}\sum_{n=1}^{\tilde{N}}X_{N}\left(n\right) \end{array}$$

(15) $$X_{N,MAD}=median(\left|X_{N}\left(n\right)-median\left(X_{N}\left(n\right)\right)\right|)$$

where $X_{N}\left(n\right)$ is the vibration signal, N is the *n*th data of the discrete wavelet frequency band (N=1,2,3,...,N), and n is the sampling data. Maximum is the maximum among all the *n*th data, and minimum indicates the minimum among all the *n*th data. Mean is the average value from the sum of the *n*th data. Range presents the influence of the difference between the maximum and the minimum from an extreme value. Standard deviation is the square root of the variance, representing the degree of discreteness of the data. Median absolute deviation concludes the data central position and is less effected by an extreme value.

### 2.4. Training and Classification of SVM, GRNN, and Deep-Learning-Based SDP

Classification is divided into two distinct steps: The first is to transform motor signals through the wavelet to compute their features. The features are thrown into SVM and GRNN for teaching and classification. The second step is to pictorialize and process the signals being transformed by the wavelet, and then to introduce the pictures into CNN for teaching and classification.

The main function of linear SVM is to find a hyperplane, which can separate the maximum margin from amongst all the input teaching data with diverse classification, where all the x located on the separating hyperplane satisfy the decision function, as shown in Equation (16):

(16) f(x)=w⋅x+b

where *w* is the normal vector of this separating hyperplane, and b is the offset. When f>0, the datum is classified as +1, and when f<0, the datum is classified as −1. When the optimal separating hyperplane is found, the support vector must satisfy Equation (17):

(17) $$y_{i}\left(x_{i}\cdot w+b\right)-1\geq 0$$

where the distance of w⋅x+b=0 is 1/‖w‖, i.e., $d_{+}=d_{-}=1/\left\|w\right\|$, and the margin is therefore 2/‖w‖. When the maximum margin of the separating hyperplane is found, it is necessary to solve the minimum of ${\left\|w\right\|}^{2}$ in compliance with Equation (17). The Lagrange Multipliers can be used to handle this optimization problem. The formula is as in Equation (18):

(18) $$L_{p}\left(w,b,\alpha \right)=\frac{1}{2}{\left\|w\right\|}^{2}-\sum_{i=1}^{n}\alpha_{i}\left[y_{i}\left(w\cdot x_{i}+b\right)-1\right]$$

where $\alpha_{i}$ is the Lagrange coefficient, and $\alpha_{i}>0$. According to the Karush–Kuhn–Tucker (KKT) Theorem, the optimum solution $\left(w_{i}^{*},b_{i}^{*}\right)$ can be substituted into Equation (18), to get Equation (19):

(19) $$\sum_{i=1}^{n}\alpha_{{}_{i}}^{*}\left[y_{i}^{*}\left(x_{i}\cdot w^{*}+b^{*}\right)-1\right]=0$$

Any $x_{i}$ satisfying Equation (19) is the answer for the support vector that is closest to the separating hyperplane. From all the above, it is possible to obtain the classification process function:

(20) $$f\left(x\right)=\mathrm{sgn}\left(\sum_{i=1}^{n}\alpha_{i}y_{i}\cdot \left(x_{i}\cdot x_{j}\right)+b\right)$$

However, not all the data are helpful for finding the linear separating hyperplane in actual practice. It is necessary to map the original data to a higher-dimensional feature space through a nonlinear mapping function, Φ, and then to implement linear classification from this feature space. If the mapping of the data is launched into a feature space, then the data will be $\Phi \left(x_{i}\right)\cdot \Phi \left(x_{j}\right)$. However, a kernel function can be used to replace $\Phi \left(x_{i}\right)\cdot \Phi \left(x_{j}\right)$, so it may not be necessary to first map the data to a feature space if the inner product of the data in the feature space can be resolved through the kernel function. The usual kernel functions include a linear, polynomial radial basis function (RBF), as well as a Sigmoid function. RBF was used in this study because the radial function is capable of sorting nonlinear data of high-dimensionality, and only two parameters need adjustment.

The very principle of GRNN is the regression of a dependent variable Y on an independent variable X, which is then described through a probability density function (PDF). This differs from traditional regression analysis, which requires the initial assumption of a definite function. If f(x,y) is the PDF for both variable X and Y, and *x* is an observed value of X, then the regression of Y at this *x* is as shown in Equation (21) [17]:

(21) $$E\left(Y|x\right)=\frac{\int_{-\infty }^{\infty }yf\left(x,y\right)dy}{\int_{-\infty }^{\infty }f\left(x,y\right)dy}$$

Since f(x,y) is still unknown, the observed values of both X and Y are needed to estimate f(x,y). The Parzen window is used to measure f(x,y), and the result is shown in Equation (22):

(22) $$\hat{f}\left(x,y\right)=\frac{1}{{\left(2\pi \right)}^{\left(p+1\right)/2}\sigma^{\left(p+1\right)}}\frac{1}{n}\times \sum_{i=1}^{n}\exp\left[-\frac{{\left(x-x^{i}\right)}^{T}\left(x-x^{i}\right)}{2\sigma^{2}}\right]\exp\left[-\frac{{\left(y-y^{i}\right)}^{2}}{2\sigma^{2}}\right]$$

where p is the dimension of x, $x^{i}$ and $y^{i}$ are the sample observed values (x,y), n is the sample size, and σ is the smoothing parameter, which is the only parameter to be configured in GRNN, and is usually between 0 and 1. We can therefore attain Equation (23) by substituting (22) into (21):

(23) $$\begin{array}{ll} \hat{E}\left(Y|x\right) & =\hat{y}\left(x\right)\\ & =\frac{\sum_{i=1}^{n}e^{\left[-\frac{{\left(x-x^{i}\right)}^{T}\left(x-x^{i}\right)}{2\sigma^{2}}\right]}\int_{-\infty }^{\infty }y\cdot e^{\left[-\frac{{\left(y-y^{i}\right)}^{2}}{2\sigma^{2}}\right]}dy}{\sum_{i=1}^{n}e^{\left[-\frac{{\left(x-x^{i}\right)}^{T}\left(x-x^{i}\right)}{2\sigma^{2}}\right]}\cdot e^{\left[-\frac{{\left(y-y^{i}\right)}^{2}}{2\sigma^{2}}\right]}dy} \end{array}$$

After simplifying Equation (23), $D_{i}^{2}={\left(x-x^{i}\right)}^{T}\left(x-x^{i}\right)$ can be set, and the estimated observed value *y* is as shown in Equation (24):

(24) $$\hat{y}\left(x\right)=\frac{\sum_{i=1}^{n}y^{i}\exp\left[-\frac{D_{i}^{2}}{2\sigma^{2}}\right]}{\sum_{i=1}^{n}\exp\left[-\frac{D_{i}^{2}}{2\sigma^{2}}\right]}$$

A Convolution Layer uses the local feature of a picture acquired by sliding from the left to the upper right, and then to the bottom through a window of fixed size. This becomes a feature as the next pooling layer; the sliding window is the convolution kernel. After the convolution kernel feature is obtained, the pooling layer is entered. The purpose of this is to lower the dimension of each feature map and retain its most important features. The pooling layer downsizes the scale of an input picture and also draws out the feature. The most usual pooling kernel scale is 2×2, and the step length is 2. There are three major pooling-layer methods: max-pooling, mean-pooling, and stochastic-pooling. The final one is a fully connected layer, a general neural network, which flattens the feature information and connects to the neural network for classification [18,19].

**Figure 6:**
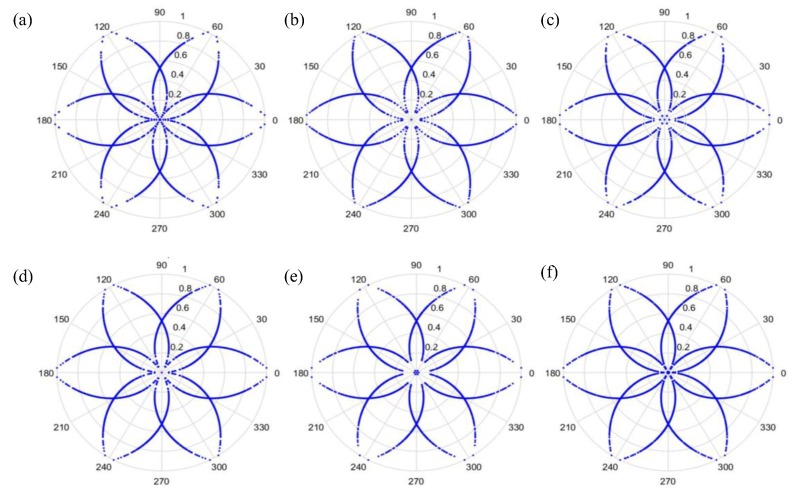
Results when t=0 and sampling is at 1000 Hz/1000 rpm. (**a**) Normal, (**b**) motor platform with one eccentric screw, (**c**) motor platform with two eccentric screws, (**d**) sanded ball bearings, (**e**) insufficient lubrication, and (**f**) worn ball bearings.

**Figure 7:**
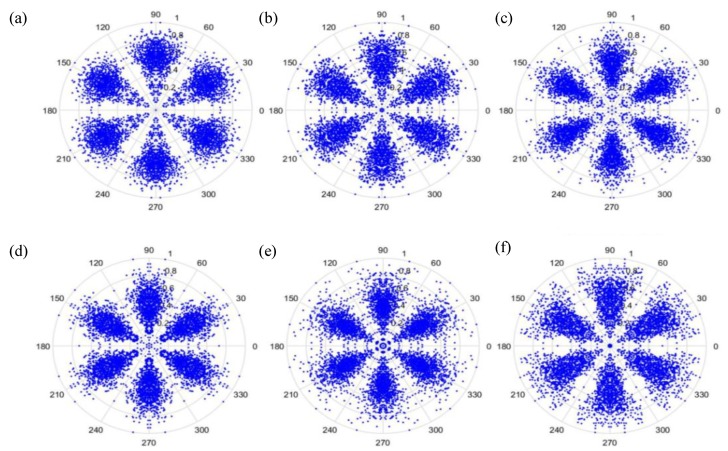
Results when t=1 and sampling is at 1000 Hz/1000 rpm. (**a**) Normal, (**b**) motor platform with one eccentric screw, (**c**) motor platform with two eccentric screws, (**d**) sanded ball bearings, (**e**) insufficient lubrication, and (**f**) worn ball bearings.

In this study, the signals were pictorialized, after which CNN was used to classify them. To do this, a map of symmetrized dots was used, as was done by Pickover [23] for the visual characterization of speech waveforms. Pickover thought that it would be rather difficult to read all the data in numeric or character form. However, if the data were represented as a graphic, with a recognizable pattern, it could be more easily appreciated and understood. The basic principle of symmetrized dot pattern (SDP) technology is the mapping of the time waveform to polar coordinates to generate a map of dots. The dots are the time waveform and are mapped to the radial component, and the angular component is mapped by adjacent dots. The transform formulas are shown from Equations (25)–(27):

(25) $$r\left(i\right)=\frac{F\left(i\right)-l}{H-L}$$

(26) $$\varphi \left(i\right)=\theta +\left(\frac{F\left(i+t\right)-L}{H-L}\right)g$$

(27) $$\varphi \left(i\right)=\theta -\left(\frac{F\left(i+t\right)-L}{H-L}\right)g$$

where F(i) is the input signal, *H* and *L* are the maximum and the minimum of F, respectively, θ is the rotational angle of the initial line, g is the signal gain, and *t* is the time-lag coefficient. When parameter *t* is changed, the outcome is shown in Figure 6 and Figure 7, when *t* is 0 or 1, respectively.

**Figure 8:**
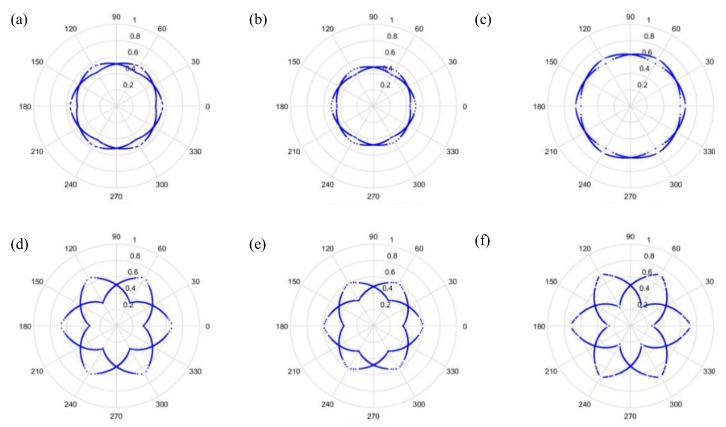
Results by improved formula when t=0 and sampling is at 1000 Hz/1000 rpm. (**a**) Normal, (**b**) motor platform with one eccentric screw, (**c**) motor platform with two eccentric screws, (**d**) sanded ball bearings, (**e**) insufficient lubrication, and (**f**) worn ball bearings.

**Figure 9:**
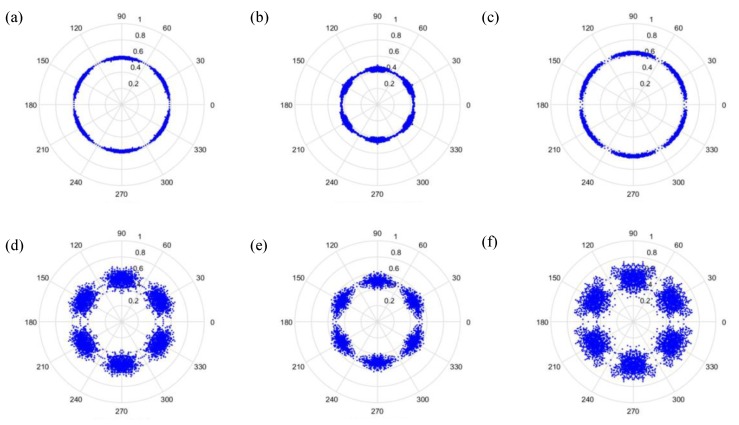
Results by improved formula when t=1 and sampling is at 1000 Hz/1000 rpm. (**a**) Normal, (**b**) motor platform with one eccentric screw, (**c**) motor platform with two eccentric screws, (**d**) sanded ball bearings, (**e**) insufficient lubrication, and (**f**) worn ball bearings.

As can be seen from Figure 6 and Figure 7, the dots are over-dispersed, and it may be difficult to spot the features of this picture via deep learning (DL). Furthermore, the maximum value, *H*, in the experimental data is between 0 and 1, and the minimum value, *L*, in the experimental data is between 0 and −1, and the answers gained from the formula L/(H−L) are located between 0 and −1. In addition, F(i) are mostly known values to the second decimal place, and, as a result, the value of F(i)−(L/(H−L)) is almost determined by L/(H−L). The signal features can be fixed within a certain scope, in order to allow the dots in the picture to concentrate and be representative of the features, to allow better CNN identification. The radius after adjustment is shown in Equation (28), and the results are shown in Figure 8 and Figure 9.

(28) $$r(i)=F\left(i\right)-\frac{L}{H-L}$$

## 3. Main Results

In modern industries, data-driven techniques are investigated by using the Internet of Things (IOT) and computer networks to gather the big-machinery data to monitor and diagnose the machine-health status [24,25]. For data-driven machine health monitoring systems (MHMS), there are three key components: feature design, feature selection and extraction, and model training. However, how to determine the suitable features is a dominant key to the MHMS. Rui Zhao et al. discussed the emerging research works of deep learning (DL) on MHMS [26]. After the extraction of the key features, some shallow machine-learning (ML) algorithms, which include SVM, Naive Bayes (NB), GRNN, and logistic regression, are usually applied to classify the status of the machinery health. However, it is difficult to determine what kind of features should be chosen for classification of the above algorithms.

Convolutional neural networks (CNNs) were proposed by LeCun for handwritten digit recognition, with two key properties, which are spatially shared weights and spatial pooling [19]. For CNN models, there are many successful cases in computer vision applications whose input data are 2D data. CNN was introduced to the sequential data, including natural language processing (NLP), speech recognition, and MHMS. For past CNN-based MHMS, the input data of CNN can be separated in two types, which are a 1D format of time sequence and a 2D format of time-frequency spectrum or two different sensors. One-dimensional CNN applied raw time series of vibration signal and the corresponding operations for fault detection. For example, Turker Ince et al. proposed an 1D CNN based on raw time-series data, to detect the motor fault, in which feature extraction and classification were integrated together [27]. In contrast to 1D-CNN-based MHMS, the 2D-CNN-based method can capture more signals from two or more sensors, to detect the motor fault. Janssens et al. used the discrete Fourier transform (DFT) of two accelerometer signals from two sensors as the input of a 2D-CNN model to recognize the four categories of rotating machinery conditions [28]. Ding et al. studied a CNN by using the input image of wavelet packet energy (WPE) for detecting spindle bearing fault [29]. Guo et al. proposed a hierarchical learning-rate-adaptive deep CNN (ADCNN); they used the input time series data as a signal-vector, and the input data were transformed into a 32 × 32 matrix, and the ADCNN model was adopted by LeNet [30]. Unlike to the above 2D-CNN models, including a transformation from the 1D input data into a 2D matrix of the CNN, the input data of the proposed method is a 2D figure for the CNN model. Abdeljaber et al. proposed 1D-CNN based on the normalized vibration signal, to detect the localization of the structural damage in real time [31]. This approach can extract optimal damage-sensitive features from the raw acceleration signals, which do not need additional preprocessing.

**Figure 10:**
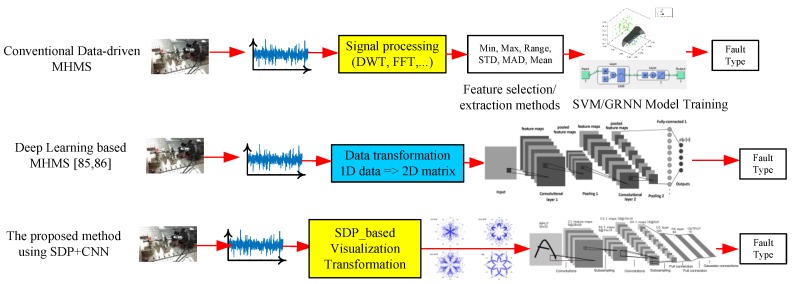
Comparison between the conventional data-driven machine-health monitoring systems (MHMS), the deep-learning-based MHMS, and the proposed method.

For obtaining the visualization of input signal, the accelerometer signals are transferred into a symmetrized dot figure to classify the different healthy status of the motor or ball bearing. In this study, the comparison between the conventional data-driven MHMS, the deep-learning-based MHMS, and the proposed method are illustrated by the principles behind these three kinds of MHMS discussed above, as shown in Figure 10. To illustrate the conventional data-driven MHMS, the SVM and GRNN methods are studied for the experimental system. For these two data-driven MHMS methods, the performance of the classification is dominated by the chosen features for classification algorithms. After discussing the experimental results of SVM and GRNN, the proposed SDP-based CNN method is illustrated for the same case, and the performance of the real-time implementation is discussed.

### 3.1. Classification Results of SVM and GRNN Methods

Some of the classified signal data collected in these experiments were used for training, and the rest were used as testing data for examining the accuracy of the classification. The classifiers selected for use were SVM and GRNN. The most suitable frequency band to make a comparison with the results of CNN was selected by testing the accuracy of two dimensions of SVM, at a sampling frequency of 1000 Hz. Then, experiments with each of the six different conditions were carried out at each frequency band. These combinations were then computed by SVM: 1000 data items in total; sampling frequency: 1000 Hz; rotational speed: 1500 rpm; frequency band: D1, D2, D3, D4, D5, and A5, and the feature method: maximum, minimum, mean, range, standard deviation, and median absolute deviation. The teaching data used were 500 data items from the first half of all the data, and the testing data were 500 data items from the second half of all the data. Table 3 describes the accuracy of classification for the SVM and GRNN, using the different selection of three features.

From Table 3, it can be seen that the degree of identification is higher for frequency bands D4 and D5, where the average identification rate on D4 is up to 95.96% when using SVM, and the rate on D5 is up to 92.79%. Furthermore, the average GRNN identification rate for D4 is 89.06% and for D5 is 97.31%. SVM clearly shows better overall stability. Moreover, the feature methods used in SVM identification here are mean, standard deviation, and median absolute deviation, and identification rates can reach 97.90%. Therefore, the features at D4 captured using mean, standard deviation, and median absolute deviation were then used to make a classification for two different frequency samples (1000 and 3000 Hz), at three different rotational speeds (1000, 1500, and 2000 rpm), in the following case studies.

**Figure 11:**
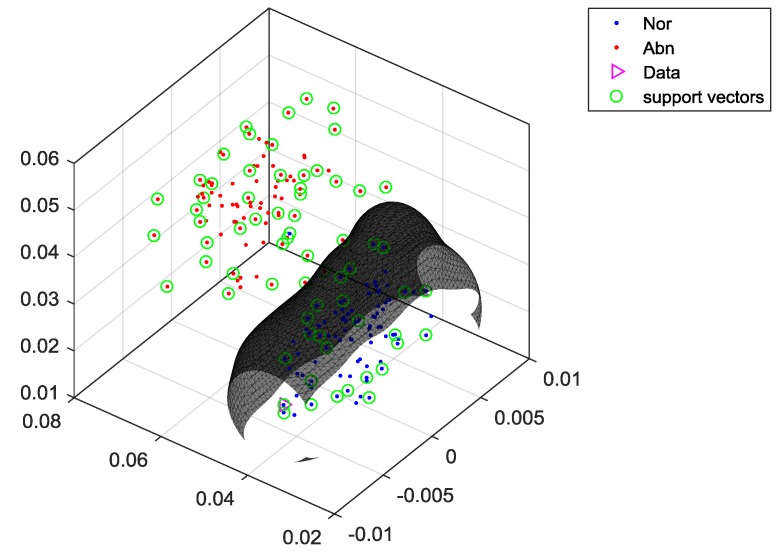
Binary tree: inspection and classification.

SVM was used for classifying the machine status, using three major testing methods. They are the internal-training (Test 1), the half-external test (Test 2), and the external test (Test 3). For the half-external test, half of the samples are used in the training of the SVM, but all the samples are used for testing. The external test represents that half of the samples are used for training, but the other half are used for testing. For the proposed SVM method, we used multistage classifiers, and there are five SVM classifiers in this case study. The first SVM classifier can separate the whole data into two groups, which are the normal state (Group 1) and the fault states (Group 2). Figure 11 shows the hyperplane of SVM, using the features (mean, standard deviation, and median absolute deviation) to separate Group 1 (the normal state) and Group 2 (the abnormal states). For the abnormal states (Group 2), there are five different faults: (F1) motor platform with one eccentric screw, (F2) motor platform with two eccentric screws, (F3) sanded ball bearings, (F4) insufficient lubrication, and (F5) worn ball bearings. The second SVM classifier separate Group 2 into Group 3 and Group 4. Group 3 represents that the motor platform with one eccentric screw. Group 4 includes four faults, which are motor platform with two eccentric screws, sanded ball bearings, insufficient lubrication, and worn ball bearings. Therefore, the first two SVM classifiers can extract out the normal (N) and the first type of fault (F1). Consequently, the third SVM classifier can separate Group 4 into Group 5 and Group 6, and it extracts the second type of fault (F2); the fourth SVM classifier can separate Group 6 into Group 7 and Group 8, and it extracts the second type of fault (F3). Finally, the fifth SVM classifier can extract F4 and F5 from Group 8. After the above classification processes, we can separate each status of the machine into six groups, as follows:

Group 1: normal state (N).
Group 3: the motor platform with one eccentric screw (F1).
Group 5: motor platform with two eccentric screws (F2).
Group 7: sanded ball bearings (F3)
Group 9: insufficient lubrication (F4).
Group 10: worn ball bearings (F5).

**Figure 12:**
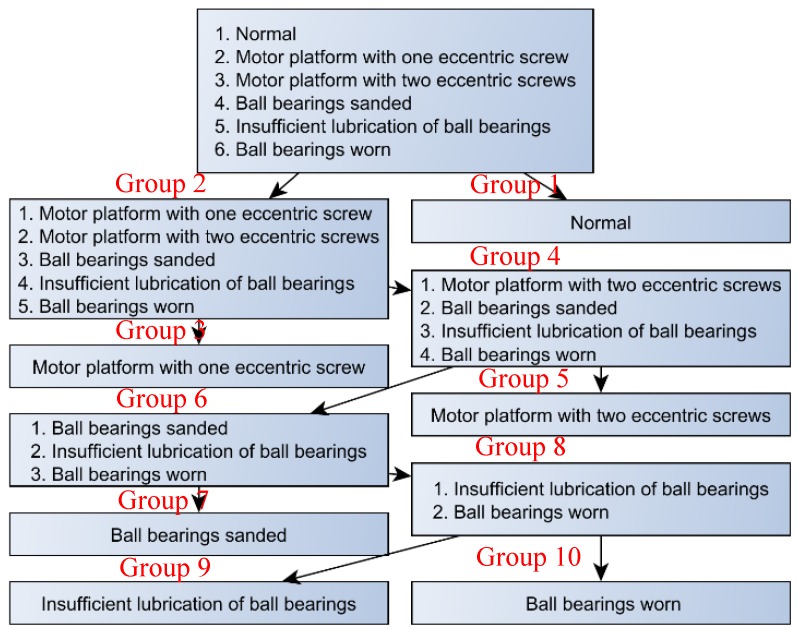
Binary tree: inspection and classification.

The classification process is shown in Figure 12, and the identification outcomes are shown in Table 4. We can observe in Table 4 that the average accuracy of SVM sampling at 3000 Hz is higher than that at 1000 Hz. This is because the higher the sampling frequency, the more apparent the captured feature, and the higher the accuracy.

### 3.2. Classification Outcomes of the Proposed SDP + CNN Method

Through the previous works, the DL-based MHMS do not require extensive human labor and expert knowledge in selecting the suitable feature functions. If the CNN is well-trained with the input data with the marked fault type, the end-to-end structure is able to map raw machinery data to the target healthy status. The DL-based MHMS can be applied to all kinds of machines with rotors and ball bearing. The DP-based MHMS with the 1D acceleration signal is influenced easily by the sensor’s noise; therefore, the SDP + CNN method is proposed to transfer the input data from the time response in a constant period to a 2D visual figure for the CNN model.

**Figure 13:**
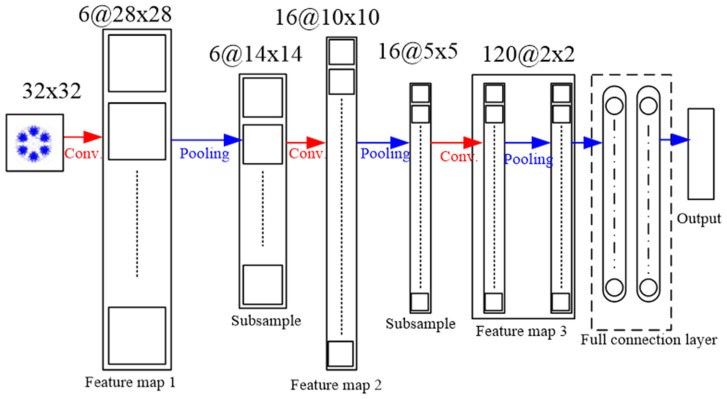
Architecture of the convolutional neural network (CNN) used for the proposed method.

The classical LeNet5 model is used for the classification of the faults based, and its architecture is shown in Figure 13. There are three layers of feature map, with a set of units whose weights must be identified. The first layer is the first feature map, which comprises a convolution layer. The second layer is the subsample layer, which uses the max-pooling operation. There are 6, 16, and 120 feature filters in the first, second, and third feature map layers, respectively. The next two layers after the three-feature map are full-connection layers, which prepare features for classification. The last layer is a logistic-regression layer, which uses the SoftMax method for classification. The weight in each layer is randomly initialed and trained for optimization. After training, the test samples are input into the logistic-regression layer, whose output comprises class labels corresponding to the samples.

**Figure 14:**
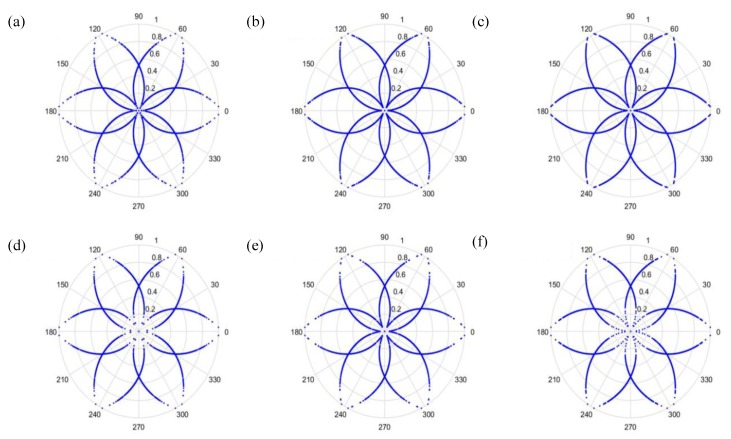
Results from improved formula when t=0 and sampling is at 3000 Hz/2000 rpm. (**a**) Normal, (**b**) motor platform with one eccentric screw, (**c**) motor platform with two eccentric screws, (**d**) sanded ball bearings, (**e**) insufficient lubrication, and (**f**) worn ball bearings.

**Figure 15:**
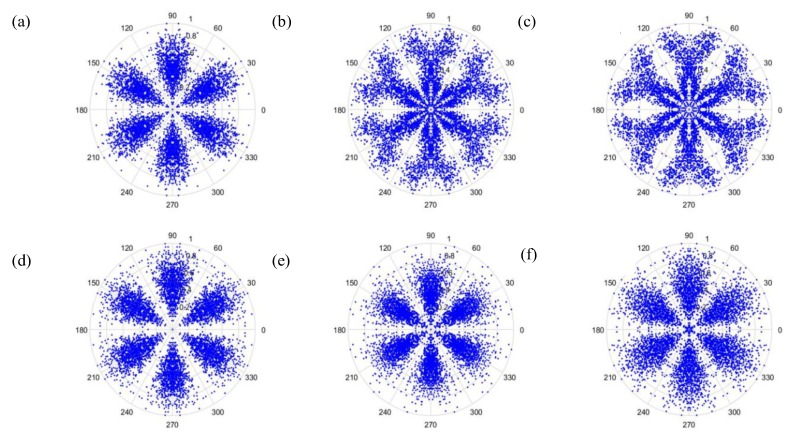
Results by improved formula when t=1 and sampling is at 3000 Hz/2000 rpm. (**a**) Normal, (**b**) motor platform with one eccentric screw, (**c**) motor platform with two eccentric screws, (**d**) sanded ball bearings, (**e**) insufficient lubrication, and (**f**) worn ball bearings.

**Figure 16:**
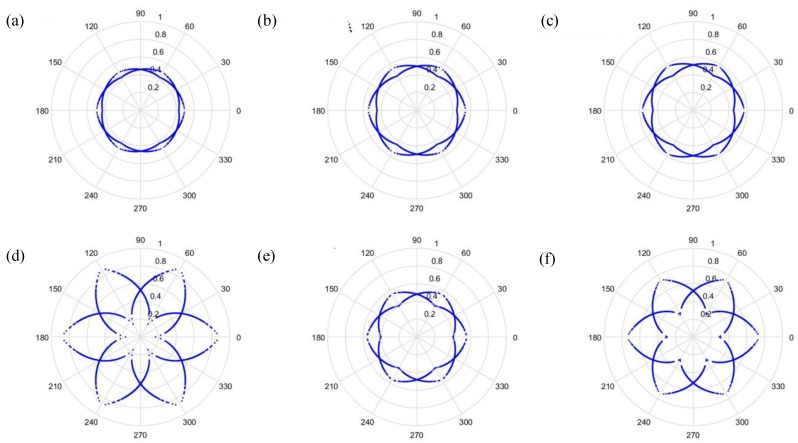
Results from improved formula when t=0 and sampling is at 3000 Hz/2000 rpm. (**a**) Normal, (**b**) motor platform with one eccentric screw, (**c**) motor platform with two eccentric screws, (**d**) sanded ball bearings, (**e**) insufficient lubrication, and (**f**) worn ball bearings.

**Figure 17:**
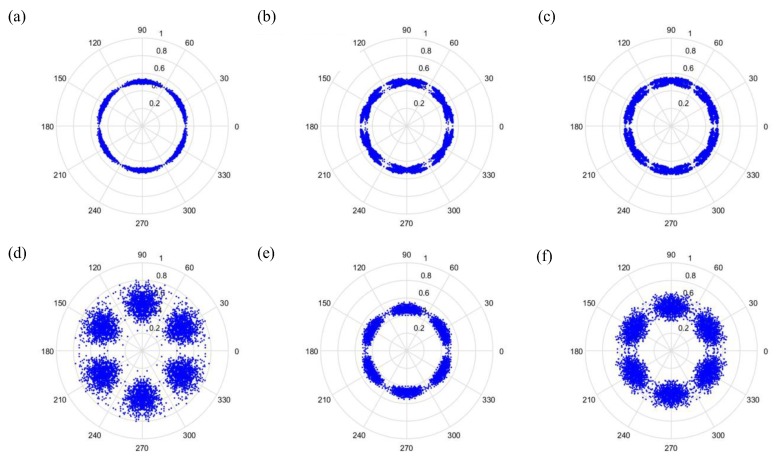
Results from improved formula when t=1 and sampling is at 3000 Hz/2000 rpm. (**a**) Normal, (**b**) motor platform with one eccentric screw, (**c**) motor platform with two eccentric screws, (**d**) sanded ball bearings, (**e**) insufficient lubrication, and (**f**) worn ball bearings.

Additionally, we studied the methods of SDP to obtain the visual image for the input of the CNN model. Figure 14 and Figure 15 show the results of the original radius formulas under the conditions of a sampling frequency and rotational speed at 3000 Hz/2000 rpm, when the time-delay is 0 or 1. Figure 16 and Figure 17 show the results of the improved radius formulas under the conditions of a sampling frequency and rotational speed at 3000 Hz/2000 rpm, when the time-delay is 0 or 1.

The most significant signal features generated at 2000 rpm were transformed into pictures and brought into CNN for teaching and classification, in order to obtain an adequate sampling frequency, radius, and time-delay coefficient. This allowed comparisons to be made between different sampling frequencies, radii, and time-delay coefficients; the results are in Table 5. It is obvious from an examination of the table that the accuracy due to the improved radius formula rose substantially. When the sampling frequency is higher, more features can be obtained, and classification is much better. When the time-delay coefficient is 1, the rotational angle of the *n*th dot will be mapped at the (*n* + 1)th dot. This means that whenever a time-delay coefficient exists, the dots in the picture will spread outward, to both the left and the right, to make the feature much clearer than can be obtained without a time-delay coefficient. The higher the sampling frequency, the more accurate the results will be, because the higher sampling frequency makes the features more obvious.

**Figure 18:**
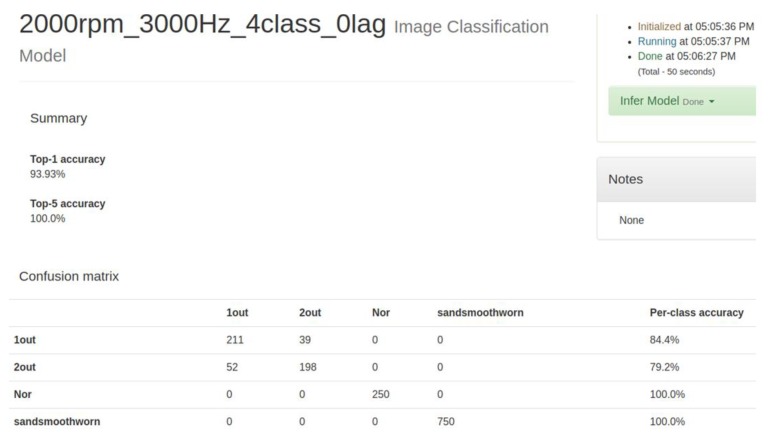
Confusion matrix for the improved formula of symmetrized dot pattern (SDP) as (sampling at 3000 Hz/2000 rpm).

**Figure 19:**
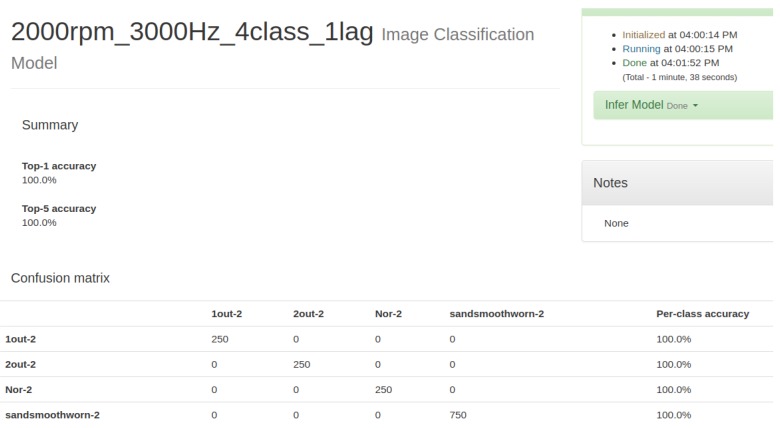
Confusion matrix for the improved formula of symmetrized dot pattern (SDP) as t=1 (sampling at 3000 Hz/2000 rpm).

An extensive set of experiments are performed using motor acceleration’s data samples, for a total of 250 healthy (H), 250 faulty with one eccentric screw (1-out), 250 faulty with two eccentric screws (2-out), and 750 faulty of the ball bearing (sand-smooth-worm) cases. The dataset is obtained from the proposed experimental platform by using the visual image of SDP transformation for capturing motor current data. The proposed SDP+CNN classifier is implemented by C++ by using MS Visual Studio 2017 in 64-bit; the CNN model is trained by using the NVidia Digits environment; and the Caffe model is exported for classification. Figure 18 and Figure 19 describe the confusion matrix for the experimental data, using the improved formula of Equation (28) as *t* = 0 and *t* = 1, respectively. In Case 1, we used the improved formula with *t* = 0 for the proposed SDP+CNN model, and its processing time of classification is about 33 ms. In Case 2, we used the improved formula with *t* = 1 for the proposed SDP+CNN model, and its processing time of classification is about 65 ms. Therefore, the possibility of the real-time implementation is guaranteed. When the improved formula of Equation (28) is used as the parameter *t* = 1 to produce the SDP image, the proposed SDP+CNN has the best classification rate. Although Case 2 has the better performance for classification than Case 1, the processing time of Case 1 is less than the one of Case 2, because the SDP image is simpler.

### 3.3. Comparison of CNN and SVM

After the optimum conditions for CNN were found, a comparison was made with those from SVM. The first conclusion reached from the six diverse classifications involved rotational speed. The experimental configuration table and results of SVM and CNN included a sampling frequency at 3000 Hz on the D4 band. The features were mean, standard deviation, and median absolute deviation. The teaching data were 500 data items from the first half of all data. The testing data were 500 data items from the second half of all data. The accuracy of SVM under different rotational speeds is shown in Table 6. The identification rates by CNN were 73.53% at 1000 rpm, 87.63% at 1500 rpm, and 94.20% at 2000 rpm. SVM was shown to be more accurate than CNN based on the experiments under the six conditions, for both rotational speed and malfunction simulation.

SVM may take longer than CNN+SDP, because finding a defect involves the classification of binary trees. However, it will be more efficient in practical application when conducting CNN + SDP because it can directly spot the classification of a defect in a picture. However, CNN also needs offline teaching.

## 4. Conclusions

This study proposes the use of an accelerometer to measure vibration signals for the diagnosis of normal and fault conditions in ball bearings. Original time-domain signals were processed by the discrete wavelet transform. These original signals were acquired from six different frequency bands from higher to lower frequency. The appropriate band was selected, the features were computed, and a classifier was chosen. The results showed that both SVM and GRNN performed well under the circumstances on the D4 band, and the feature value was (average + standard deviation + median absolute deviation), as well as with two types of classification.

A comparison showed SVM performed better than GRNN; however, the key features of the measure signals need manual operations to determine based on the experimental database, such as Table 3. It is unsuitable and time-consuming for the current industrial applications. Therefore, it is more convenient to understand what is the current status of the machine with obtaining the visualization of input signal. In this study, the accelerometer signals were transferred into a symmetrized dot figure, in order to classify the different health statuses of the motor or ball bearing. The results also showed that the time-delay coefficient had a very strong effect on the resulting picture which was to be used for CNN teaching and classification. The accuracy of classification was also influenced by the time-delay coefficient. Classification by SVM takes longer than with CNN + SDP, because finding a defect involves the use of binary trees. However, the proposed CNN + SDP method also needs offline training. This study offers real-time capture and analysis for the determination of the health of ball bearings under actual running conditions, but the DL program is implemented by using the PC architecture with the GPU card. In the future, if the embedded system is used to speed up the computing performance, it can reduce the inspection time and satisfy the demand for intellectualization.

## Figures and Tables

**Table 1:** Hardware model.

Types of Hardware	Model
Data Capture Cards	NI myRIO
Controller	CX5140
Servomotor	AM8121-0F10
Servo Motor Driver Module	EL7211
Cable wire (Servomotor Use)	ZK4704-0421-2000

**Table 2:** The db4 motor frequency bands in respondence.

1000 Hz		3000 Hz	
Hierarchy	Frequency (Hz)	Hierarchy	Frequency (Hz)
D1	500~1000	D1	1500~3000
D2	250~500	D2	750~1500
D3	125~250	D3	375~750
D4	62.5~125	D4	187.5~375
D5	32.25~62.5	D5	93.75~187.5
A5	0~32.25	A5	0~97.75

**Table 3:** Accuracy of three dimensions by Support Vector Machine (SVM) and Generalized Regression Neural Network (GRNN).

Feature	Classifier	D1	D2	D3	D4	D5	A5
Max, Min, and Mean	SVM	68.10	67.10	80.80	91.90	91.20	72.20
	GRNN	59.60	55.20	64.30	88.90	97.00	58.40
Max, Min, and Range	SVM	64.20	61.60	71.50	90.20	90.20	70.90
	GRNN	60.80	55.90	64.10	88.90	96.90	67.30
Max, Min, and STD	SVM	68.80	72.20	75.70	97.70	94.10	71.20
	GRNN	59.50	54.90	64.80	90.80	97.60	59.60
Max, Min, and MAD	SVM	70.10	70.30	77.20	96.50	92.70	72.90
	GRNN	59.60	55.20	65.00	90.50	97.90	58.60
Max, Mean, and Range	SVM	68.70	65.00	80.50	91.30	90.90	73.00
	GRNN	59.60	56.20	63.50	87.40	97.00	68.80
Max, Mean, and STD	SVM	69.80	73.30	81.30	97.80	94.00	71.20
	GRNN	56.10	56.70	60.80	84.20	96.90	60.80
Max, Mean, and MAD	SVM	68.40	70.80	80.00	95.70	92.40	68.40
	GRNN	56.20	56.70	61.00	83.70	97.10	60.10
Max, Range, and STD	SVM	68.00	71.20	74.40	97.70	94.20	71.20
	GRNN	59.50	56.30	63.80	87.90	97.10	69.10
Max, Range, and MAD	SVM	69.30	68.10	75.60	96.30	92.70	72.70
	GRNN	59.60	56.10	63.70	88.10	97.40	69.10
Max, STD, and MAD	SVM	70.60	70.40	77.00	97.60	93.60	73.00
	GRNN	56.10	56.80	61.80	87.30	97.90	61.00
Min, Mean, and Range	SVM	67.50	65.70	80.20	91.40	90.70	72.00
	GRNN	59.50	54.50	65.40	89.30	96.90	65.30
Min, Mean, and STD	SVM	72.40	70.80	82.70	97.80	93.60	71.40
	GRNN	59.00	51.10	63.00	88.20	97.20	59.80
Min, Mean, and MAD	SVM	70.60	71.00	81.50	96.20	92.30	68.80
	GRNN	59.20	50.80	63.10	87.00	97.50	59.30
Min, Range, and STD	SVM	67.80	70.40	75.00	97.60	93.50	70.90
	GRNN	59.50	54.30	65.30	90.00	97.00	66.30
Min, Range, and MAD	SVM	67.30	68.60	77.30	96.20	92.70	72.50
	GRNN	59.50	54.50	65.50	89.90	97.10	65.60
Min, STD, and MAD	SVM	70.50	70.30	76.20	97.60	93.50	72.80
	GRNN	59.00	51.00	63.40	90.90	98.00	59.60
Mean, Range, and STD	SVM	69.00	71.80	82.30	97.70	93.20	71.70
	GRNN	60.10	56.00	64.30	89.80	97.30	69.30
Mean, Range, and MAD	SVM	70.10	71.80	81.80	96.40	92.80	73.60
	GRNN	60.10	55.80	64.30	89.80	97.40	69.20
Mean, STD, and MAD	SVM	71.30	71.20	82.70	97.90	93.80	72.40
	GRNN	48.00	54.40	51.10	98.00	97.50	65.10
Range, STD, and MAD	SVM	71.70	71.10	74.40	97.60	93.70	71.80
	GRNN	60.10	56.10	64.00	90.50	97.60	69.30

**Table 4:** Accuracy of multiple SVM classification by six malfunction simulations.

1000 rpm	Test 1		Test 2		Test 3	
Sampling Rate	1000 Hz	3000 Hz	1000 Hz	3000 Hz	1000 Hz	3000 Hz
Group 1 and Group 2	78.25	66.82	77.45	67.38	71.70	68.67
Group 3 and Group 4	82.30	83.34	81.52	83.86	76.72	81.84
Group 5 and Group 6	96.05	95.80	94.15	96.33	91.80	99.80
Group 7 and Group 8	99.07	100.00	97.87	99.47	96.27	98.93
Group 9 and Group 10	100.00	100.00	99.70	96.55	99.40	93.10
1500 rpm	Test 1		Test 2		Test 3	
Group 1 and Group 2	88.67	91.68	89.47	92.17	86.87	91.20
Group 3 and Group 4	98.92	96.68	96.56	88.62	94.24	83.24
Group 5 and Group 6	100.00	97.53	99.35	97.18	98.70	97.60
Group 7 and Group 8	80.30	98.47	79.17	97.07	77.13	95.40
Group 9 and Group 10	100.00	100.00	99.40	98.35	98.80	96.70
2000 rpm	Test 1		Test 2		Test 3	
Group 1 and Group 2	86.60	99.93	84.68	99.90	84.17	99.93
Group 3 and Group 4	99.98	98.90	99.62	97.92	99.28	97.64
Group 5 and Group 6	100.00	99.98	99.58	99.93	99.15	99.90
Group 7 and Group 8	95.50	99.63	94.97	98.73	93.80	97.87
Group 9 and Group 10	100.00	96.55	99.45	93.70	98.90	91.80

**Table 5:** Comparison of different sampling frequency and time-delay coefficients.

	Original Radius Formula		Improved Radius Formula	
Sampling Rate	1000 Hz	3000 Hz	1000 Hz	3000 Hz
Time lag = 0	35.20	50.13	82.27	89.07
Time lag = 1	43.07	55.13	84.47	94.20

**Table 6:** Accuracy results of SVM at different rotational speeds.

Revolution(s) Per Minute (rpm)/SVM	1000 (rpm)	1500 (rpm)	2000 (rpm)
Group 1 and Group 2	68.67	91.20	99.93
Group 3 and Group 4	81.84	83.24	97.64
Group 5 and Group 6	99.80	97.60	99.90
Group 7 and Group 8	98.93	95.40	97.87
Group 9 and Group 10	93.10	96.70	91.80
AVG	88.47	92.83	97.43

## References

[1] Ferreira F.I., de Aguiar P.R., Lopes W.N., Martins C.H.R., Ruzzi R.D., Bianchi E.C., D’Addona D.M. (2019). Inferential measurement of the dresser width for the grinding process automation. Int. J. Adv. Manuf. Technol..

[2] Zapico P., Patino H., Valino G., Fernandez P., Rico J.C. (2019). CNC centralized control for digitizing freeform surfaces by means of a conoscopic holography sensor integrated in a machining centre. Precis. Eng. J. Int. Soc. Precis. Eng. Nanotechnol..

[3] Hoff K.A., Bashir M. (2015). Trust in automation: Integrating empirical evidence on factors that influence trust. Hum. Factors.

[4] Wang S.Y., Wan J.F., Zhang D.Q., Li D., Zhang C.H. (2016). Towards smart factory for industry 4.0: A self-organized multi-agent system with big data based feedback and coordination. Comput. Netw..

[5] Zheng J.D., Pan H.Y., Yang S.B., Pan Z.W. (2017). Generalized variational mode decomposition and its applications to gearbox fault diagnosis under variable conditions. J. Vib. Eng..

[6] Choi S., Pazouki E., Baek J., Bahrami H.R. (2015). Iterative Condition Monitoring and Fault Diagnosis Scheme of Electric Motor for Harsh Industrial Application. IEEE Trans. Ind. Electron..

[7] Buono D., Siano D., Frosina E., Senatore A. (2017). Gerotor pump cavitation monitoring and fault diagnosis using vibration analysis through the employment of auto-regressive-moving-average technique. Simul. Model. Pract. Theory.

[8] Van Loan C. (1992). Computational Frameworks for the Fast Fourier Transform.

[9] Adamczak S., Makiela W., Stepien K. (2010). Investigating Advantages and Disadvantages of the Analysis of a Geometrical Surface Structure with the Use of Fourier and Wavelet Transform. Metrol. Meas. Syst..

[10] Goupillaud P., Grossmann A., Morlet J. (1984). Cycle-octave and related transforms in seismic signal analysis. Geoexploration.

[11] Mallat S.G. (1989). A theory for multiresolution signal decomposition: The wavelet representation. IEEE Trans. Pattern Anal. Mach. Intell..

[12] Daubechies I., Combes J.-M., Grossmann A., Tchamitchian P. (1989). Orthonormal bases of wavelets with finite support—connection with discrete filters. Wavelets.

[13] Jain A.K., Duin R.P.W., Mao J.C. (2000). Statistical pattern recognition: A review. IEEE Trans. Pattern Anal. Mach. Intell..

[14] Smola A.J., Scholkopf B. (2004). A tutorial on support vector regression. Stat. Comput..

[15] Vapnik V.N. (1999). An overview of statistical learning theory. IEEE Trans. Neural. Netw..

[16] Specht D.F. Probabilistic neural networks for classification, mapping, or associative memory. Proceedings of the IEEE 1988 International Conference on Neural Networks.

[17] Specht D.F. (1991). A general regression neural network. IEEE Trans. Neural. Netw..

[18] Mallat S. (2016). Understanding deep convolutional networks. Philos. Trans. A Math Phys. Eng. Sci..

[19] LeCun Y., Bengio Y., Hinton G. (2015). Deep learning. Nature.

[20] Zhu X., Hou D., Zhou P., Han Z., Yuan Y., Zhou W., Yin Q. (2019). Rotor fault diagnosis using a convolutional neural network with symmetrized dot pattern images. Measurement.

[21] Zhu X., Zhao J., Hou D., Han Z. (2019). An SDP Characteristic Information Fusion-Based CNN Vibration Fault Diagnosis Method. Shock Vib..

[22] Allen J.B., Rabiner L.R. (1977). A unified approach to short-time Fourier analysis and synthesis. Proc. IEEE.

[23] Pickover C.A. (1986). On the use of symmetrized dot patterns for the visual characterization of speech waveforms and other sampled data. J. Acoust. Soc. Am..

[24] Yin S., Li X., Gao H., Kaynak O. (2015). Data-Based Techniques Focused on Modern Industry: An Overview. IEEE Trans. Ind. Electron..

[25] Jeschke S., Brecher C., Meisen T., Özdemir D., Eschert T. (2017). Industrial internet of things and cyber manufacturing systems. Industrial Internet of Things.

[26] Zhao R., Yan R., Chen Z., Mao K., Wang P., Gao R.X. (2019). Deep learning and its applications to machine health monitoring. Mech. Syst. Signal Process..

[27] Ince T., Kiranyaz S., Eren L., Askar M., Gabbouj M. (2016). Real-Time Motor Fault Detection by 1-D Convolutional Neural Networks. IEEE Trans. Ind. Electron..

[28] Janssens O., Slavkovikj V., Vervisch B., Stockman K., Loccufier M., Verstockt S., Van de Walle R., Van Hoecke S. (2016). Convolutional Neural Network Based Fault Detection for Rotating Machinery. J. Sound Vib..

[29] Ding X., He Q. (2017). Energy-Fluctuated Multiscale Feature Learning with Deep ConvNet for Intelligent Spindle Bearing Fault Diagnosis. IEEE Trans. Instrum. Meas..

[30] Guo X., Chen L., Shen C. (2016). Hierarchical adaptive deep convolution neural network and its application to bearing fault diagnosis. Measurement.

[31] Abdeljaber O., Avci O., Kiranyaz S., Gabbouj M., Inman D.J. (2017). Real-time vibration-based structural damage detection using one-dimensional convolutional neural networks. J. Sound Vib..

